# OMEX metadata specification (version 1.2)

**DOI:** 10.1515/jib-2021-0020

**Published:** 2021-10-20

**Authors:** John H. Gennari, Matthias König, Goksel Misirli, Maxwell L. Neal, David P. Nickerson, Dagmar Waltemath

**Affiliations:** University of Washington, Seattle, USA; Humboldt University, Berlin, Germany; Keele University, Newcastle, UK; Seattle Children’s Research Institute, Seattle, USA; Auckland Bioengineering Institute, The University of Auckland, Auckland, New Zealand; University of Greifswald, Greifswald, Germany

**Keywords:** biosimulation modeling, COMBINE standards, metadata, semantics

## Abstract

A standardized approach to annotating computational biomedical models and their associated files can facilitate model reuse and reproducibility among research groups, enhance search and retrieval of models and data, and enable semantic comparisons between models. Motivated by these potential benefits and guided by consensus across the COmputational Modeling in BIology NEtwork (COMBINE) community, we have developed a specification for encoding annotations in Open Modeling and EXchange (OMEX)-formatted archives. This document details version 1.2 of the specification, which builds on version 1.0 published last year in this journal. In particular, this version includes a set of initial model-level annotations (whereas v 1.0 described exclusively annotations at a smaller scale). Additionally, this version uses best practices for namespaces, and introduces omex-library.org as a common root for all annotations. Distributing modeling projects within an OMEX archive is a best practice established by COMBINE, and the OMEX metadata specification presented here provides a harmonized, community-driven approach for annotating a variety of standardized model representations. This specification acts as a technical guideline for developing software tools that can support this standard, and thereby encourages broad advances in model reuse, discovery, and semantic analyses.

**Author contribution:** All authors have accepted responsibility for the entire content of this manuscript and approved its submission.
**Research funding:** This work was funded in part by the NIH grant P41 GM109824 (JHG, DPN MLN) and by the Federal Ministry of Education and Research (BMBF, Germany), grant number 031L0054 (KM) and by the DFG (Germany) grant number 436883643 (KM).
**Conflict of interest statement:** Authors state no conflict of interest.

***Corresponding author: John H. Gennari**, University of Washington, Seattle, USA, E-mail: gennari@uw.edu. https://orcid.org/0000-0001-8254-4957
**Matthias König**, Humboldt University, Berlin, Germany. https://orcid.org/0000-0003-1725-179X
**Goksel Misirli**, Keele University, Newcastle, UK
**Maxwell L. Neal**, Seattle Children’s Research Institute, Seattle, USA. https://orcid.org/0000-0002-2390-6572
**David P. Nickerson**, Auckland Bioengineering Institute, The University of Auckland, Auckland, New Zealand. https://orcid.org/0000-0003-4667-9779
**Dagmar Waltemath**, University of Greifswald, Greifswald, Germany. https://orcid.org/0000-0002-5886-5563

## 1 Introduction

### 1.1 Motivation

Metadata annotations enhance the interoperability, reusability, comparability, and comprehension of computational models in biology. Annotations can capture the biological meaning of what a model simulates, specify precisely the components comprising a model, describe a model's provenance, provide layout information for visualizing a model's architecture, etc. These annotations can be leveraged to make it easier for researchers to find and re-purpose models, re-combine models and model parts, and integrate models across repositories and experimental data stores. For example, semantic annotations can be leveraged to enhance model search capabilities by identifying models that overlap in terms of the biological phenomena they represent.

Realizing the potential benefits of annotation requires the development of standards that adhere to a community-based annotation protocol. Without such standards, researchers must account for a variety of annotation formats and approaches, a situation that can become prohibitively cumbersome and which can defeat the purpose of annotating a model.

This document was created to specify how to represent model annotations within the Open Modeling and EXchange (OMEX) file format [1]. Our goal is to harmonize and simplify the representation of metadata annotations for models that are shared among the biological research community regardless of a model's encoding format. Although the focus of this document is a description of annotations for computational models, the same mechanisms can and should be applied for all assets within an OMEX archive (e.g. simulation descriptions or data sets). Our hope is that community-wide adherence to this specification will significantly advance the community's ability to discover relevant models and data sets as well as to re-purpose/re-combine models and model components.

#### 1.1.1 Cross-format search

Researchers cannot easily search across model repositories for models that simulate a particular biological process. As illustrated by Henkel, et al. [2], if the annotations on models in various repositories were encoded according to a common standard, this would make it easier to develop tools for searching across repositories and modeling formats.

#### 1.1.2 Semantic similarity between models

Using standardized metadata annotations to capture the biological properties simulated by a model allows developers to quantify how similar two models are in terms of the biological phenomena they represent (see, for example, [3, 4]). Such objective measures of biological similarity are critical for developing tools that help users discover related models within and across model repositories, exposing users to new models that may be relevant to their research.

#### 1.1.3 Semantics-based composition

Thorough semantic annotations on models are also critical for performing semantics-based model composition. This compositional approach, which aims to reduce the time and code-level edits required to merge models into larger systems, leverages machine-readable semantic annotations to automatically propose biologically-consistent interfaces between models [5]. The use of a consistent annotation protocol is necessary for achieving this level of composability for biological models.

#### 1.1.4 Semantic integration of empirical data and simulation models

Given that models are largely intended to reproduce, explain, and predict empirical data measurements, any general solution for annotating model elements would also be applicable for annotating empirical data used for model parameterization and validation. For example, the same semantic annotation on a CellML model variable that represents aortic blood pressure could be used to annotate an empirical aortic blood pressure measurement recorded in a data file. Using a common, standardized approach for annotating models as well as empirical data would accelerate the development of tools that help modelers discover data sets of interest for model parameterization or validation and help experimentalists discover models of interest for use in analyses.

## 2 OMEX Metadata technical specification

This section presents the technical specification for associating metadata with the contents of COMBINE archives [1] in the OMEX file format.

### 2.1 Conventions used in this document

Resource Description Framework (RDF, https://www.w3.org/RDF/) content and in-paragraph references to RDF subjects, predicates, and objects are indicated by `typewriter font`. We use Turtle (Terse RDF Triple Language) serialization in this document; however, other formats (e.g., RDF/XML) are equivalent.

For namespaces, we use the following prefixes for Uniform Resource Identifiers (URIs) of recommended knowledge resources and standards:

```
@prefix rdf:   <http://www.w3.org/1999/02/22-rdf-syntax-ns#> .
@prefix foaf:  <http://xmlns.com/foaf/0.1/> .
@prefix dc:    <http://purl.org/dc/terms/> .
@prefix orcid: <https://orcid.org/> .

@prefix bqmodel:     <http://biomodels.net/model-qualifiers/> .
@prefix bqbiol:      <http://biomodels.net/biology-qualifiers/> .
@prefix pubmed:      <https://identifiers.org/pubmed:> .
@prefix NCBI_Taxon:  <https://identifiers.org/taxonomy:> .
@prefix biomod:      <https://identifiers.org/biomodels.db:> .
@prefix chebi:       <https://identifiers.org/CHEBI:> .
@prefix uniprot:     <https://identifiers.org/uniprot:> .
@prefix obp:         <https://identifiers.org/opb:> .
@prefix fma:         <https://identifiers.org/FMA:> .
@prefix semsim:      <http://bime.uw.edu/semsim/> .
```

We also use a special namespace to indicate entities defined locally within the annotation file or within the OMEX archive. Some annotations will need to make reference to local entities, and these are indicated (for example) as "local:entity123". The OMEX archive itself consists of several files, which also need to be referred to in a unique manner. To refer to a specific archive, we will assume a base location of `<http://omex-library.org/>`. URIs with this base will not resolve (omex-library.org is an empty website), but this base provides a unique address for identifying all models in a single RDF graph. In addition, this base name supports a longer-term vision where there is a distributed library of OMEX archives. Such a library would need some method to ensure unique archive names, but this task is beyond our current scope of work.

For example, we use the following as a prefix for a single model (the code file):

```
@prefix OMEXmodel: <http://omex-library.org/ArchiveName.omex/ModelName.ext#> .
```

In the above, "ModelName.ext" should be replaced by something that is uniquely identifying and specific, such as "chang_fujita_1999.cellml" or "BIOMD00000345.sbml". Likewise, local entities defined within the rdf annotation file can be defined as:

```
@prefix local: <http://omex-library.org/ArchiveName.omex/ModelName.rdf#> .
```

All prefixes are simply shorthand (reducing the size of the rdf file), and tools can choose to serialize annotations without these prefixes. In addition, this format is shorthand specifically for the Turtle syntax; in RDF/XML syntax, these URIs would have to written out longhand.

### 2.2 Concepts used in OMEX Metadata

#### 2.2.1 COMBINE archives

A COMBINE archive (also known as an OMEX archive) is a single zip file containing the various documents necessary for the description of a model as well as all associated data and simulation procedures. These documents include, for example, simulation experiment descriptions, all models needed to run the simulations, associated data files, etc. The archive is encoded using the OMEX format. Version 1 of the OMEX specification is available at https://co.mbine.org/specifications/omex.version-1.

#### 2.2.2 RDF

The Resource Description Framework (RDF) is a World Wide Web Consortium-recommended standard for representing information on the Web. RDF consists of statements built using subject-predicate-object triples that can be used to assert relationships between model components and terms from online knowledge resources. A primer on RDF is available at https://www.w3.org/TR/rdf11-concepts/, and links to examples of RDF-encoded model annotations can be found in this document.

#### 2.2.3 Model-level annotations

A model-level annotation is an annotation that captures an aspect of the model *as a whole*. Examples include an annotation that indicates the PubMed ID of the model's source publication, an annotation that indicates that the model simulates the glycolysis pathway, or an annotation that indicates the identity of the person who encoded the model.

#### 2.2.4 Archive-level annotations

Archive-level annotations are metadata items that capture information about the archive as a whole. These may be especially important when the archive includes multiple models. These annotations can also be used to explain relationship across multiple files–e.g., how or why a SED-ML file captures a particular simulation result from the model.

#### 2.2.5 Model-component annotations

A model-component annotation is a metadata item that captures, entirely or in part, the meaning of a model component. The model component must be identifiable via a metadata identifier (metaid, see below) in the source code. By "model component", we mean fine-grained constituents of a model, rather than entire sub-models. Thus, model-component annotations include those that are about (a) physical properties (such as those that might be encoded by CellML variables), (b) physical entities (such as "species" in SBML), and (c) processes, such as biochemical reactions. For example, an annotation on a model variable might indicate that it represents the concentration of cytosolic glucose in a pancreatic beta cell (a physical property). Separate annotations might indicate the biochemical entity of glucose and the anatomic entity of pancreatic beta cells. Model-component annotations can also be for purely computational features such as the simulation time-step.

#### 2.2.6 Singular annotations

Singular annotations are those that are comprised of a single RDF statement linking a model or data element to a knowledge resource term. These are the types of annotations currently found throughout curated models on BioModels. See section 2.3.6 for examples.

#### 2.2.7 Composite annotations

Composite annotations are semantic annotations that are comprised of multiple annotation terms linked using standard qualifiers (also known as "relations" or "predicates") to indicate the meaning of an annotation. Composite annotations are used when a single knowledge resource term is not available to sufficiently define a model or data element. For annotations on model components, composite annotations have two primary elements: the physical property represented by the annotated item (e.g., chemical concentration, fluid volume) and the physical entity, process, energy differential, or dependency that bears the property (e.g., a pool of ATP in the cytoplasm, blood in a cardiac cavity, the glucokinase reaction). See section 2.3.7 for examples.

#### 2.2.8 identifiers.org URIs identifiers.org [6] is a resolving system that enables referencing of data for the scientific community with a focus on the life sciences domain. It handles persistent identifiers in the form of URIs and Compact URIs (CURIEs). identifiers.org also provides standardized URI prefixes for a large set of biological knowledge resources.

#### 2.2.9 BioModels.net qualifiers

BioModels.net qualifiers are a set of standardized relations (also known as "predicates") used to indicate the nature of the relationship between an annotation and its annotated element or between components of an annotation. For example, the BioModels.net qualifier `is` is used to indicate the identity of an annotated element and the qualifier `isEncodedBy` is used to indicate that a particular protein is encoded by a particular DNA sequence.

#### 2.2.10 Metadata identifiers

In standardized XML-based model exchange formats such as the Systems Biology Markup Language (SBML [7] [8]), CellML [9], NeuroML [10] and the Simulation Experiment Description Markup Language (SED-ML [11]), the XML elements often have an attribute for specifying a metadata ID. For example, the following SBML code from model BIOMD0000000001 on BioModels.net indicates that the model has metadata ID "_000001".

```
<model metaid="_000001" id="BIOMD0000000001" name="Edelstein1996 - EPSP ACh event">
...
</model>
```

These metadata IDs are unique to each XML element within a XML document and are used in annotation statements to link each annotation to the corresponding XML element that they describe.

### 2.3 Serializing OMEX Metadata

This specification for serializing annotations in OMEX-formatted documents is based largely on the article by Neal et al. [12], which presents a list of recommendations for standardizing semantic annotations on biological models. This part of the specification addresses how to standardize the storage of annotations within COMBINE archives, an essential technical prerequisite for harmonizing their representation across model annotation efforts. For simplicity, this document assumes one is annotating a single model; however, the approach should scale to multiple models, and to models that have sub-models as components.

#### 2.3.1 Serialization format

We recommend encoding OMEX metadata in RDF. RDF has emerged as the *de facto* standard for encoding annotations among the COMBINE community, and all COMBINE standards currently use it. Although more expressive knowledge representation formats exist, RDF is sufficiently expressive for articulating the kinds of annotations required to catalyze significant advances in model discovery, reuse and integration. We recommend using RDF content that is formatted as RDF/XML because this is the format most widely supported by software libraries. However, annotations can be formatted as Turtle (https://www.w3.org/TR/turtle/) or *n*-triples (`https://www.w3.org/TR/n-triples/`) as well. For readability, we use Turtle in this document. Software that supports reading/writing COMBINE archive annotation files should support these alternative formats in addition to RDF/XML.

#### 2.3.2 Separation of annotations from models and data

We recommend using separate RDF files to store all annotations associated with model and simulation protocol files within a COMBINE archive. The traditional practice within the COMBINE community has been to serialize annotations within the same file that specifies the model's computational aspects. However, more recently the community has agreed that storing annotations separately from code is preferred [12]. There are several reasons why we recommend storing annotations in a separate file. First, this will normalize the format in which annotations are stored across the different COMBINE standards. Currently, the exact format used to store annotations within model files differs among standards. Normalizing the format will simplify the development of software that provides programmatic manipulation of annotations. It will also allow for better separation between modeling and annotation tasks, removing the burden of supporting annotation from the software teams that are developing software libraries for specific COMBINE standards.

We also recommend storing annotation files separately because we recognize that different research groups may have different preferences for which knowledge resources to use for annotation. Externalizing annotations in a separate file allows a single model file to be referenced by multiple annotation files, allowing different research groups to describe the same modeling resource in different ways. This approach follows the vision of the COMBINE archive, wherein multiple types of modeling files are archived together to make simulation experiments readily reproducible and shareable among research groups. When sharing models, we recommend that annotations be distributed along with the files they annotate, and COMBINE archives provide a standardized way to bundle such files together. An additional advantage of storing annotations in a separate file is that the RDF content can be serialized in various formats, including XML or Turtle. Currently, the serialization is dictated by the model format.

Storing annotations in a separate file requires keeping them synchronized. For example, if a variable identifier changes in the model file, that change should be reflected in the annotation file(s) as well. We recommend that the community encourages the development of software libraries and tools that help ensure coordination between a model's computational aspects and its annotations.

Multiple RDF annotation files are allowed within an archive and the OMEX manifest file should provide sufficient information so that parsers can automatically determine which files within an archive contain the RDF annotations. Software tools should provide support for reading the content of each individual annotation file into separate RDF graphs and, alternatively, for reading the content of multiple files into one merged RDF graph.

#### 2.3.3 Formatting URIs in RDF

The subjects of RDF triples used for annotation should include the name of the file to be annotated, and the metadata ID of the annotated element within the file as the URI fragment. As described above in the discussion of namespaces, we use the "OMEXmodel" prefix to uniquely identify the metadata IDs within the model code of a specific OMEX archive. For example, if there is a model file in that archive and it contains an element with metadata ID "meta0", then the subject URI used in an annotation statement on that model element would be:

```
OMEXmodel:meta0
```

Broadly, COMBINE archive annotations should leverage existing ontology resources to describe information about the model. For example, we build directly from the Dublin Core Metadata initiative, especially for authorship and provenance information about a model. Likewise, wherever possible, COMBINE archive annotation documents should use BioModels.net qualifiers in RDF statements that define model elements. These existing qualifiers provide a basic level of coverage needed for articulating annotations in models, and they are specifically intended for use in statements that link computational abstractions of physical phenomena to knowledge resource terms representing the material manifestations of those phenomena.

In addition to BioModels.net qualifiers to encode singular annotations, SemSim qualifiers should be used in composite annotations to unambiguously encode the relationships between the annotation's components (see 2.3.7). As illustrated in the examples in 2.3.7, SemSim qualifiers are primarily used to indicate physical entity participation in a physical process or energy differential as well as the stoichiometry of a process's participants.

When available, we recommend using the identifiers.org URI format when referencing knowledge resource terms in RDF statements: identifiers.org supports a vast set of biological knowledge resources used for annotation and identifiers.org-formatted URIs are resolvable. The identifiers.org services are also capable of more complex URI resolution compared to alternative services. For example, identifiers.org is specifically built to address downtime and changing endpoints and directs users to an alternative site for a given data record as long as one is listed (one-to-many mappings) whereas persistent uniform resource locator services specify only one endpoint for URI resolution (one-to-one mappings). We also recommend using identifiers.org-formatted URIs because they use a simple, uniform nested structure that facilitates generation and parsing, and because identifiers.org reuses data providers' record identifiers.

In this document, we show RDF examples using the Turtle (terse RDF triple language) syntax; this is equivalent to RDF/XML, but is more human-readable (see https://www.w3.org/TR/turtle/).

#### 2.3.4 Serializing model-level annotations

An important model-level annotation is the "author" of the model. For this idea, we leverage the Dublin Core notion of "creator". However, this apparently simple idea can rapidly become complex. For example, there may be an "author" of the publication of the model, who did not actually produce the model code. There may also be "authors" of the specifications of particular executions (with particular parameter values) of a model for a particular result (these might be specified in SED-ML files). Finally, there may be "curators" who add or edit a model's annotations.

For this iteration of the metadata specification, we take an intentionally simple approach, following the lead of Dublin Core. In that ontology, there are just two terms that cover the range of authorship: "creator" and "contributor". Anyone with the "creator" tag is a person who is responsible for the creation of the model code file (e.g. the SBML or CellML model code). This may be more than one person and may or may not include the primary author of the manuscript/publication describing the model. If model developers wish to indicate others who have edited, fixed, or augmented the model, then they may use the dc:contributor tag. However, in most cases we expect that the dc:creator tag will provide sufficient detail. For creators or contributors, the model-level annotation must be linked to the metadata ID for the <model> tag in the source code. Thus, if an SBML model has metadata ID "model01", then authorship could be indicated by:

```
OMEXmodel:model01 dc:creator orcid:0000-0001-8254-4957 .
```

If we wish to indicate a curator or editor of the model, we could say:

```
OMEXmodel:model01 dc:contributor orcid:0000-0002-2390-6572 .
```

In the above, the agent is indicated by an ORCID identifier. This identifier is unique, and should point to additional information about the person. However, if the annotator wishes, for improved readability, to include additional information (such as a string with the creator's name), then they can provide that information via additional triples and foaf relations:

```
orcid:0000-0002-2390-6572 foaf:name "John Smith" ;
                          foaf:mbox <mailto:jsmith63@fakegmail.com> .
```

If authors (or contributors) do not have an orcid, then they must be identified by "local" information:

```
OMEXmodel:model01 dc:creator local:author01 .
local:author01 foaf:name "John Smith" ;
               foaf:mbox <mailto:jsmith63@fakegmail.com> .
```

Including authorship, developers must support at least the following types of model-level annotations:

| | |
|---|---|
| dc:creator | An author of the model |
| dc:contributor | An editor or curator of the model |
| dc:created | The date (timestamp) when the model was created |
| dc:description | Free text providing a title or description of the model |
| bqmodel:isDescribedBy | The publication associated with the model |
| bqmodel:isDerivedFrom | Provenance information |
| bqbiol:hasTaxon | The taxon (or species) that the model is intended for |

As can be seen, these annotation types are taken from Dublin Core (creator, created, etc.) as well as from the biomodels qualifiers. The "isDerivedFrom" annotation can provide simple provenance information, such as an indication of other models that were precursors to this model. Ideally, such an annotation would point to other models in models repositories such as the CellML library or the BioModels collection.

Finally, "hasTaxon" indicates the biological entity (e.g., species) that the model is designed for, or possibly the species from which data was collected to build the model. Thus, there may be more than one hasTaxon, as in the example below about avian influenza. The following block shows how these model-level annotations could be used.

```
OMEXmodel:model01
            dc:creator orcid:0000-0001-8254-4957 ;
            dc:created "2018-07-18"^^dc:W3CDTF ;
            dc:description "Dynamics of avian influenza with Allee growth effect" ;
            bqmodel:isDescribedBy pubmed:27887851 ;
            bqmodel:isDerivedFrom biomod:BIOMD0000000279 ;
            bqbiol:hasTaxon NCBI_Taxon:9606 ;
            bqbiol:hasTaxon NCBI_Taxon:8782 .
```

Library developers that aim to support this version of the specification must allow for at least the 7 types of model-level annotation shown in the table above. However, in general, we allow for any of the qualifiers specified by Biomodels.net.

#### 2.3.5 Serializing archive-level annotations

At present, most OMEX archives consist of a single model, an annotation file for that model, and possibly a SEDML file that describes initial settings for a specific simulation. For these sort of archives, we expect that there will be minimal need for archive-level annotations. In these common situations, the archive author ("dc:creator") and the archive date of creation ("dc:created") should be sufficient.

For now, "dc:description" could be used for free-text description of the purpose of the OMEX archive, e.g., any specific results tables from particular publications that the simulation should be able to produce. In the future, with multi-model archives, additional annotation may be needed to describe the relationship among these models.

#### 2.3.6 Serializing model component singular annotations

Singular annotations within COMBINE archive annotation files should be encoded as a single RDF triple. The subject of the triple is a URI referring to the annotated element. The predicate is the URI of a BioModels.net qualifier linking the subject to a URI from a knowledge resource or the Dublin Core Metadata Terms qualifier `description`. The object of the triple should be an identifiers.org-formatted URI indicating a concept in a knowledge resource, or a text string for free-text definitions of model elements.

The following is an example singular semantic annotation indicating that the model element with metadata ID "meta0013" from the model file "MyModel.sbml" represents adenosine triphosphate:

```
OMEXmodel:meta0013 bqbiol:is chebi:15422 .
```

The following is an example free-text description of a model variable with metadata ID "meta0014":

```
OMEXmodel:meta0014  dc:description  "Cardiomyocyte cytosolic ATP concentration" .
```

#### 2.3.7 Serializing model-component composite annotations

Composite annotations are used to capture the biological meaning of model or data elements when no singular reference term is available that provides a complete definition. Based on the SemSim framework [13], composite annotations have two parts: the *physical property* that is represented, and what it is a property *of*. The second component, the bearer of the property, is either a physical entity, process, energy differential or dependency.

##### *2.3.7.1 Composite annotation for a property of a physical entity*

Consider a CellML variable that simulates blood volume in the left coronary artery. The physical property simulated is volume; more precisely, *fluid* volume. This fluid volume is a property of blood in the lumen of the left coronary artery. Because there is no existing knowledge resource term that represents "blood volume in the left coronary artery", we instead construct a composite annotation using a combination of existing knowledge resource terms. For the physical property portions, we recommend using terms from the Ontology of Physics for Biology (OPB [14]) because the OPB provides a comprehensive, formally-structured hierarchy of physical properties. In this case, we would use the OPB term "Fluid volume" (opb:OPB_00154). The second part of the composite annotation, blood in the left ventricle, can be created by linking two terms from the Foundational Model of Anatomy (FMA [15]), namely "Portion of blood" (FMA:9670) and "Lumen of left coronary artery" (FMA:18228). We link these two FMA terms using the `isPartOf` BioModels.net qualifier to produce a composite physical entity. Thus, the composite annotation links the model element being annotated to the physical property it represents (via the `isVersionOf` BioModels.net qualifier) as well as to the composite physical entity that bears the property (via the `isPropertyOf` BioModels.net qualifier).

Encoded as RDF (turtle format), this example would be serialized as:

```
OMEXmodel:VLV bqbiol:isVersionOf opb:OPB_00154  ;
              bqbiol:isPropertyOf local:entity_0 .

local:entity_0 bqbiol:is fma:9670 ;
               bqbiol:isPartOf fma:18228 .
```

Figure 1 shows a node and edge diagram for the example composite annotation:

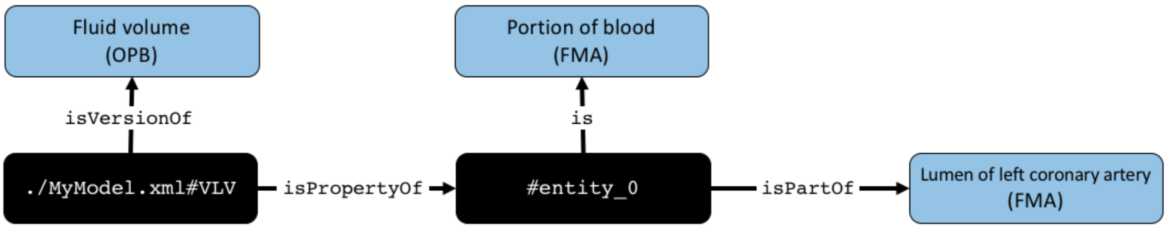

Figure 1. Node and edge representation of an example composite annotation for a model variable that simulates blood volume in the left coronary artery. RDF resources local to the OMEX metadata file are in black boxes, external knowledge resource terms are in blue boxes.

Note that the local RDF resource `entity_0` needs to be created when encoding the composite annotation. This is required because there are no structures in the CellML schema that represent physical entities, so they are instantiated as RDF resources in the OMEX metadata document. Such instantiation is often not needed for SBML models, since SBML provides explicit XML elements for representing the bearers of physical properties including chemical species, compartments and reactions, and these elements can have unique metadata IDs assigned to them.

However, instantiation of a resource that represents the full concept described by the composite annotation *is* needed for the properties of species, compartments, and reactions in SBML models. Although SBML uses XML structures for declaring physical entities (<compartment> and <species> elements) as well as processes (<reaction> elements), it does not declare XML elements that represent the *properties* of those entities and processes. Instead, the property is indicated by an XML attribute on the entities and processes. Therefore, to ensure that these properties are searchable in OMEX annotations, we recommend instantiating a generic RDF resource for each physical property that is implicitly represented in an SBML model. Note that this does not have to be done when annotating an SBML <parameter> because these elements includes a metadata ID that can be explicitly referenced in the OMEX metadata file.

##### 2.3.7.2 *Composite annotation for a property of a physical process*

The example annotation above is for a physical property of a physical entity. However, models also represent the rates of physical processes, such as chemical reactions, transport of solutes, flow of fluids in vessels, etc. For models at the subcellular level, biochemical reactions are a critical part of many models.

When modeling these sorts of reactions, researchers face a choice. From a mathematical perspective, the elements of concern are the reaction flow rates, the equations that govern these, and resulting impacts on chemical concentrations of participants. In this view, the flow rates are named with variables, and the equations can be used to compute changes in concentrations over time. An example of this approach is built into the CellML modeling language, where reactions are only identified by named flow rates.

In contrast, one could choose to name the reaction itself by, for example, listing its participants or providing a unique identifier for the process itself. In this scheme, the mathematical equations that govern the reaction rate could be implicit, or could be fulfilled by different mathematics, corresponding to different assumptions. An example of this approach is built-in to the SBML language, where the reaction equation is implicit.

As our annotation approach is agnostic to such choices, we must support both choices. It is critical to note that reactions are *processes*, whereas reaction flow rates are *properties* of those processes. Thus, regardless of modeling language, our annotations include the process, its participants, and its properties.

Therefore, for processes, we use a composite annotation where a custom physical process is instantiated and linked to its participants: the energetic sources and sinks as well as mediators whose amounts modulate the magnitude of the flow rate. Sources, sinks and mediators are the physical entities that participate in the process: source amounts are consumed by the process, sink amounts are produced, and mediator amounts remain unchanged. In the biochemical domain, sources and sinks correspond to "reactants" and "products", but the terms "source" and "sink" are broader in scope. For a reaction, the biochemical flow rate is a property of the process.

Below, we provide an example of a composite annotation for the rate of a chemical reaction with one source, one sink, and one mediator. Initially, we present this in the style of CellML, where the flow rates are named in the model source code.

First, we assert statements indicating that the model includes a variable that represents a physical property of a process that is a chemical flow rate (opb:OPB_00592).

```
OMEXmodel:property_metaid_0
     bqbiol:isPropertyOf local:process_0 ;
     bqbiol:isVersionOf opb:OPB_00592 .
```

In the above, `property_metaid_0` points to the CellML variable representing the chemical flow rate.

We next assert statements that indicate the physical entity participants in the process: the energetic sources, sinks, and mediators. In this case, there is one source, one sink, and one mediator. Optionally, the sources and sinks can include stoichiometry statements (using the `semsim:hasMultiplier` qualifier) to indicate the production/consumption ratios for the process participants (mediator participation statements cannot include stoichiometries). RDF statements indicating the biological identity of the chemical species that participate in the process (the resources with the `species_metaid_*` URI fragments in this example) would be included elsewhere in the RDF.

```
local:process_0
       semsim:hasSourceParticipant local:source_0 ;
       semsim:hasSinkParticipant local:sink_0 ;
       semsim:hasMediatorParticipant local:mediator_0 ;
       bqbiol:isVersionOf <https://identifiers.org/GO:0004022> .
local:source_0
       semsim:hasMultiplier 1.0 ;
       semsim:hasPhysicalEntityReference OMEXmodel:species_metaid_0 .
local:sink_0
       semsim:hasMultiplier  2.0 ;
       semsim:hasPhysicalEntityReference OMEXmodel:species_metaid_1 .
local:mediator_0
       semsim:hasPhysicalEntityReference OMEXmodel:species_metaid_2 .
```

In the above, the process is identified as "isVersionOf" a particular GO:process term. However, often an appropriate knowledge resource term that precisely represents a modeled process may not be available, and thus this sort of triple is optional. Lacking a GO term or a Rhea or EC number (for metabolic reactions), biological processes must simply be identified by the process participants (as enumerated above).

If the modeler is using SBML, or any language that expects reactions or processes to be explicitly named in the source code, the annotation would look a bit different. The differences are in which entities are defined locally (with `local:` prefixes) versus which have pointers back to the source code (with `OMEXmodel:` prefixes). For SBML, the flow rate is local, and the process has a metadata ID:

```
local:property_0
     bqbiol:isPropertyOf OMEXmodel:reaction_metaid_0 ;
     bqbiol:isVersionOf  opb:OPB_00592 .
OMEXmodel:reaction_metaid_0
     semsim:hasSourceParticipant local:source_0 ;
     semsim:hasSinkParticipant local:sink_0 ;
     semsim:hasMediatorParticipant local:mediator_0 ;
     bqbiol:isVersionOf <https://identifiers.org/GO:0004022> .
local:source_0
     semsim:hasMultiplier 1.0 ;
     semsim:hasPhysicalEntityReference OMEXmodel:species_metaid_0 .
local:sink_0
     semsim:hasMultiplier  2.0 ;
     semsim:hasPhysicalEntityReference OMEXmodel:species_metaid_1 .
local:mediator_0
     semsim:hasPhysicalEntityReference OMEXmodel:species_metaid_2 .
```

Notice that in either case, the annotations about the sources, sinks and mediators are the same.

For SBML, we recognize that creating composite annotations for biological processes in this manner can duplicate information that is present in the structure of SBML reactions, which includes information about reactants and products, etc. However, because these annotations are stored independently from the source code, these composite annotations are necessary so that all biological features represented in a model are exposed in the RDF metadata. This way, the community can more easily apply RDF-processing tools to analyze, query, and reason over semantic metadata in COMBINE archives, in a manner that is independent of the source code used by the model.

##### 2.3.7.3 Composite annotations for energy differentials (e.g., pressures & voltages)

Composite annotations can also be used to represent the properties of energy differentials. These include, for example, membrane potentials, chemical potentials and fluid pressures. The structure of annotations for these properties is similar to process properties. Because energy differentials are not conventionally named or represented explicitly in model code, we use a local resource in the RDF with its energetic sources and sinks specified. (Mediators and stoichiometries are only used for process annotations.)

The following is an example that represents the electrical potential caused by a difference in the amount of charged ions on either side of a cell membrane. For this example, we assume a model element with metadata ID “parameter_metaid_0” represents this biological property. We assert triples stating that the model element represents a property of an energy differential, and specifically that it represents a voltage (opb:OPB_01058):

```
OMEXmodel:parameter_metaid_0
      bqbiol:isPropertyOf local:EnergyDiff_0 ;
      bqbiol:isVersionOf opb:OPB_01058 .
```

We add triples that indicate the physical entity participants (energetic sources and sinks) whose properties define that energy difference:

```
local:EnergyDiff_0
   semsim:hasSourceParticipant :source_23 ;
   semsim:hasSinkParticipant :sink_12 .
local:source_23
   semsim:hasPhysicalEntityReference OMEXmodel:species_metaid_42 .
local:sink_12
   semsim:hasPhysicalEntityReference OMEXmodel:species_metaid_37 .
```

For SBML models, the URI fragments `species_metaid_42` and `species_metaid_37` would correspond to metadata IDs on <species> elements in the SBML code (species such as intra- and extra-cellular calcium ions, for example). Importantly, for models in CellML or other formats that do not include the explicit representation of physical entities, these metadata IDs would point to local physical entity resources instantiated elsewhere in the RDF metadata.

##### 2.3.7.4 Composite annotation for a property of a physical dependency

The final type of physical properties that are represented in simulation models are properties of physical dependencies. Also known as "constitutive properties", these include, for example, electrical resistance, fluid volumetric elastance, and reaction rate constants. Unlike entity, process, and energy differential properties, dependency properties characterize the mathematical relationship between *two or more* physical properties. For example, linear electrical resistance is defined as the slope of the relationship relating electrical potential across a resistor and the electrical current through it.

Currently, we advise annotators to only indicate the represented physical property for model elements that quantify these types of properties. We believe it is sufficient to say that a certain model parameter or variable "is an electrical resistance" rather than encode all information about the physical properties that play a role in the dependency. To determine which specific physical properties in the model are related through the dependency (and thus, which physical entities, processes and/or energy differentials), software libraries should be able to examine the equation solving for the constitutive property and then identify which physical role players are involved in the dependency based on the identifiers used in the equation.

#### 2.3.8 Annotating tabular data

Biomedical data is often stored and shared in plain-text, delimited tables organized into rows and columns. Annotating these tables using OMEX metadata would provide a way to describe the data in more detail than the plain-text format provides. Therefore, we recommend that OMEX metadata libraries provide basic support for serializing and retrieving annotations on tabular data files within COMBINE archives. While more sophisticated strategies may emerge with time, for now we recommend that libraries support annotating individual columns within tabular data files using the column header as a surrogate metadata ID that is referenced in annotation statements within the OMEX metadata file. For example, if a data file contains a column with values that represent the volume of blood in the left coronary artery recorded over time, the meaning of the data in the column could be captured using the composite annotation example in section 2.3.7.1. The composite annotation would look the same as in the example, except the URI for the subject of the first statement would refer to a data file (within the OMEX archive) and a column header ("VleftCorArt" in the RDF below):

```
<http://omex-library.org/ModelName.OMEX/MyData.csv#VleftCorArt>
  bqbiol:isVersionOf opb:OPB_00154 ;
  bqbiol:isPropertyOf local:entity_67 .

local:entity_67
  bqbiol:is fma:9670 ;
  bqbiol:isPartOf fma:18228 .
```

Note that this strategy for annotating data requires column headers to be unique within a data file. We encourage the community to continue to develop strategies for annotating data in commonly-used formats.

#### 2.3.9 Annotating physical units

Currently, the COMBINE community does not have a consensus, cross-format approach for annotating the meaning of physical units declared in models or for indicating the physical units on experimental data values. We recommend that the community develop strategies to support physical unit annotation so that software tools can easily recognize unit mismatches when comparing semantically equivalent model elements (for example, during model merging) or when associating model variables/parameters with experimental data.

### 2.4 Knowledge resources to use for annotation

Different members of the modeling community may prefer to use different ontologies or databases when annotating their models and associated files. Therefore, software packages that adhere to this specification should allow annotators to use a wide variety of knowledge resource terms. However, in the interest of introducing a degree of standardization to the annotation process, we provide the following set of recommended knowledge resources. Note that this list primarily focuses on annotation of models and does not address other file types (SED-ML, for example) that may be packaged in COMBINE archives.

#### 2.4.1 Resources to use for model-level annotations

The specific knowledge resources used for model-level annotations largely depend on the curatorial objectives of model development teams. Thus, we cannot make comprehensive recommendations about which resources should be used for these annotation types. However, to link a model to its source publication, we recommend using the publication's PubMed ID or DOI. To indicate the taxon for which the model is applicable, we recommend using identifiers from the NCBI taxonomy resource. To indicate the physical bounds within which a model's simulated phenomena occur, we recommend applying a singular model-level annotation that uses the `bqbiol:occursIn` relation to link the model to a term from, for example, one of the physical entity knowledge resources listed in the following section.

#### 2.4.2 Resources to use for composite semantic annotations

The SemSim development group makes the following recommendations for which knowledge resources to use when creating a composite semantic annotation (CSA):

**Physical *property* component of a CSA**

- Ontology of Physics for Biology (OPB)

**Physical *entity* component of a CSA**

- Chemical Entities of Biological Interest (ChEBI) for atoms and small molecules (e.g., metabolites)
- Protein Ontology (PR) for proteins
- UniProt can also be used to annotate proteins in a model when it is important to disambiguate the proteins based on their amino acid sequence or taxonomic species
- Gene Ontology:cellular component for subcellular structures
- Cell Type Ontology (CL) for cell types
- Foundational Model of Anatomy (FMA) for structures at the tissue scale or higher
- Mouse Adult Gross Anatomy (MA) ontology for rodent-specific gross anatomy
- Ontology for Biomedical Investigations (OBI) for laboratory materials

**Physical *process* component of a CSA**

Physical processes are always defined using a custom (*ad hoc*) term; however, `bqbiol:isVersionOf`, `bqbiol:isPartOf` and `bqbiol:hasPart` statements can be added to the custom term to give it semantic context. The recommended knowledge resources to use for those statements include:

- Gene Ontology, the biological process branch
- Rhea database, for biochemical reactions (especially metabolic reactions)

#### 2.4.3 Internally referencing biological knowledge stored within COMBINE archives

Depending on their research domain, some modelers may find that external knowledge resource terms that sufficiently disambiguate elements in their model are unavailable. In such instances we recommend making term requests to maintainers of the appropriate knowledge resources so that reference terms needed by the community are added to those resources. We also recommend that software packages supporting this specification provide functions and services to expedite these requests.

However, for researchers in some modeling domains, the annotation terms needed to disambiguate model elements may require a level of detail not supported among current biomedical knowledge resources. Thus, some researchers may need to compose complete descriptions of their own knowledge resource terms to define a model element. Since these descriptions may not be aggregated into publicly-available collections, and thus may not possess web-resolvable URIs, they should be storeable within COMBINE archives so they can be referenced in OMEX annotation statements.

For example, this issue arises in modeling protein modifications such as phosphorylation. A complete collection of knowledge resource terms that circumscribe all possible protein phosphorylations is unavailable, and may be untenable in terms of maintenance. This issue may also arise in synthetic biology models where model elements are disambiguated by nucleotide or amino acid sequences, and where non-canonical biopolymers are represented.

We therefore recommend that OMEX annotation software packages support annotation statements that link an annotated item to an *ad hoc* knowledge term stored within the COMBINE archive. For example, an annotator should be able to reference a nucleotide or amino acid sequence in a FASTA file contained in the archive in order to link, say, a non-canonical protein in an SBML model to the sequence description that defines it. Any encoded knowledge items stored within the OMEX file should include a unique identifier (e.g., those used in the headers of FASTA file entities) so that they can be used in RDF annotation statements linking models and/or data elements to knowledge items within the archive.

RDF statements that reference an internal knowledge term should do so using a URI composed of the path to the file in the archive followed by the unique identifier for the item. For example, the URI for an entry with the unique identifier "seq1" in a top-level FASTA file named "MySeqs.fasta" should be

```
<http://omex-library.org/ModelName.OMEX/MySeqs.fasta#seq1>
```

OMEX annotation software libraries should interpret the fragment in these URIs as an entry in the unique identifier field of whatever format the knowledge item is stored in. For a FASTA file, the fragment would indicate the text in the header/identifier field of a FASTA entry. Other formats for storing knowledge terms might include the Synthetic Biology Open Language (SBOL [16]) , BioPax [17], BpForms [18], BcForms [18], the Web Ontology Language (OWL, https://www.w3.org/TR/owl2-overview/, or the Open Biomedical Ontology (OBO) format [19].

### 2.5 OMEX Metadata resources

Community discussions on OMEX metadata issues can be found at the combine-annot Google group: https://groups.google.com/forum/#!forum/combine-annot.

A C/C++ library to support this specification has been developed at https://github.com/sys-bio/libOmexMeta

Python bindings for this package can be found at https://pypi.org/project/pyomexmeta/

Information on the COMBINE archive format can be found at http://co.mbine.org/standards/omex

## 3 Acknowledgements

This specification has been developed with the valuable input of Daniel Cook, Jonathan Cooper, Andreas Dräger, Alan Garny, Nick Juty, Jonathan Karr, Chris Myers, and Herbert Sauro.

#### 3.0.1 Author contributions

All authors (editors) have accepted responsibility for the entire content of this manuscript and approved of its submission.

#### 3.0.2 Research funding

This work was funded in part by the NIH grant P41 GM109824 (JHG, DPN MLN) and by the Federal Ministry of Education and Research (BMBF, Germany), grant number 031L0054 (KM).

## A The COMBINE archive

A COMBINE archive is a single file containing the various documents (and in the future, references to documents), necessary for the description of a model and all associated data and procedures. This includes for instance, but is not limited to, simulation experiment descriptions in SED-ML, all models needed to run the simulations in SBML and their graphical representations in SBGN-ML. It is a convenient alternative if a model source URI cannot be resolved, or if an end-user is offline.

The SED-ML archive described in appendix D of the SED-ML Level 1 Version 1 specification formed the basis for the COMBINE archive with contributions from the SED-ML and COMBINE communities.

The COMBINE archive is described at: `https://co.mbine.org/documents/archive`.
